# Characterization of oral polymorphonuclear neutrophils in periodontitis patients: a case-control study

**DOI:** 10.1186/s12903-018-0615-2

**Published:** 2018-08-24

**Authors:** Elena A. Nicu, Patrick Rijkschroeff, Eva Wartewig, Kamran Nazmi, Bruno G. Loos

**Affiliations:** 1 Department of Periodontology, Academic Centre for Dentistry Amsterdam (ACTA), University of Amsterdam and VU University Amsterdam, Gustav Mahlerlaan 3004, 1081 LA Amsterdam, The Netherlands; 2 Opris Dent SRL, Sibiu, Romania; 3 Department of Oral Biochemistry, Academic Centre for Dentistry Amsterdam (ACTA), University of Amsterdam and VU University, Amsterdam, The Netherlands

**Keywords:** Periodontitis, Polymorphonuclear neutrophilic granulocytes, Reactive oxygen species, Apoptosis, Degranulation

## Abstract

**Background:**

Maintaining oral health is a continuous and dynamic process that also involves the immune system. Polymorphonuclear neutrophils (PMNs) migrate from blood circulation and become apparent in the oral fluid. Controversies exist regarding the specific role of the oral PMNs (oPMNs) in the presence of chronic oral inflammation, such as periodontitis. In this study we characterized cell counts, activation status, apoptosis, and reactive oxygen species (ROS) generation by oPMNs and circulatory (cPMNs), and the salivary protease activity, in subjects with and without periodontitis.

**Methods:**

Venous blood and oral rinse samples were obtained from 19 patients with untreated periodontitis and 16 control subjects for PMN isolation. Apoptosis and expression of cell activation markers CD11b, CD63, and CD66b were analyzed using flow cytometry. Constitutive ROS generation was detected using dihydrorhodamine123. Additionally, ROS production in response to stimulation was evaluated in samples incubated with 10 μM phorbol myristate acetate (PMA) or *Fusobacterium nucleatum*. Total protease activity was measured using substrate PEK-054.

**Results:**

Periodontitis patients presented with over 4 times higher oPMN counts compared to controls (*p* = 0.007), which was a predictor for the total protease activity (*r*^*2*^ = 0.399, *P* = 0.007). More oPMNs were apoptotic in periodontitis patients compared to the controls (*P* = 0.004). All three activation markers were more expressed on the oPMNs compared to the cPMNs (*p* < 0.05), and a higher expression of CD11b on the oPMNs from periodontitis patients was observed compared to the control subjects (*P* = 0.024). Constitutive ROS production per oPMN was higher compared to the cPMN (*P* < 0.001). Additional analysis showed that the oPMNs retained their ability to respond to stimulation, with no apparent differences between the periodontitis and control subjects.

**Conclusions:**

Higher numbers of oral PMNs, being more apoptotic and having increased levels of degranulation markers were found in periodontitis compared to periodontal health. However, since the oPMNs in periodontitis were responsive to ex vivo stimulation, we conclude that the oPMNs are active in the oral ecosystem. It is currently unknown whether the oPMN counts, which correlated with the detected protease levels, are detrimental in the long term for the oral mucosa integrity.

**Trial registration:**

This study was retrospectively registered at the ISRCTN registry (trial ID ISRCTN15252886). Registration date August 11, 2017.

## Background

Oral health is often confused with the absence of oral disease. However, oral health is a dynamic state, maintained as long as the active equilibrium between oral microbiota, salivary defense mechanisms, and host immune responses exists [1, 2]. Despite the heavy colonization with oral microorganisms, overt infections rarely occur in the oral cavity.

Polymorphonuclear neutrophils (PMNs) are primary determinants in the host response to microbes. They constitute the most abundant population of white blood cells, and are capable of recognizing, binding, internalizing and killing microorganisms [3]. PMNs are recruited to inflammatory sites and are crucial for the clearance of infection. The majority of the existing studies focused on PMNs retrieved from venous blood, which may not necessarily reflect the PMNs’ functional contribution on the mucous membranes within the oral cavity. Importantly, the current knowledge is mainly derived from studying PMNs in diseased states, and little is known about their contribution to oral health maintenance [4–10].

Previously, our group characterized PMNs in the oral cavity (oPMNs) from a large group of systemically and orally healthy young individuals [11]. There, we established that on average 1.0 × 10^6^ oPMNs can be purified after 4 rinses, and the PMNs were capable of reactive oxygen species (ROS) production in response to bacterial stimulation ex vivo. We also studied the oPMN in edentulous subjects and found that in the absence of teeth, the number of oPMNs was significantly decreased [12]. Moreover, the oPMNs in edentulous individuals were basically non-functional, being either apoptotic or impaired and not capable of responding to bacterial stimulation ex vivo. The absence of functional oPMNs might compromise the resilience of the edentulous oral cavity.

In a healthy mouth, low numbers of PMNs constantly migrate through the oral epithelia and become apparent in the oral fluid. The sulci around the teeth form an important source for oPMNs; for a long time it has been known that with gingival inflammation the number of oPMNs is increased [4, 7, 13]. Interestingly, Dutzan et al. characterized PMNs in biopsies of both gingiva and buccal mucosa. They concluded that the PMN numbers were correlated with the levels of chemotactic factors in the tissues and found higher PMN numbers in periodontitis patients compared to non-periodontitis at both of these locations [14].

Periodontitis is a chronic inflammatory disease of the supporting tissues of the teeth, initiated in susceptible subjects by an aberrant immune response to dental plaque, and exacerbated by a dysbiotic biofilm. Although the PMN’s primary role is protective against microbial invasion, continuous recruitment and migration of PMNs through the periodontal tissues might contribute to the collateral damage and tissue breakdown in periodontitis [15]. Our knowledge about the PMNs that have “travelled” from the gingiva into the periodontal pocket, along the biofilm, into the oral cavity is limited and it is not known whether the oPMN in periodontitis patients can still contribute to oral health maintenance. Older studies only reported on oPMN numbers in periodontitis [13, 16], while more recent studies showed the possibility to study the phenotype and functional capabilities of oPMNs [6, 8, 17, 18]. The oPMNs may represent a distinct subset of peripheral PMNs, which acquire specific traits in periodontitis patients.

To gain more knowledge on the role of oPMNs in periodontitis, this study aimed to characterize the oPMNs numbers and function, in patients with untreated periodontitis and compare those to cells from subjects without periodontitis. Since all oPMNs are migrated cells from the peripheral blood circulation, we also included the analysis of circulatory PMNs (cPMNs) to investigate possible changes of PMN features that may occur when PMNs journey from blood into the oral cavity.

## Methods

The study was approved by the Medical Ethical Committee of the *VU* University Medical Center, The Netherlands (2012–210#B2012406, March 29th 2013) and conducted in accordance to the Declaration of Helsinki [19]. All subjects were informed about the purpose of the study, received written information and had given written consent prior to inclusion. This study was retrospectively registered at the ISRCTN registry (trial ID ISRCTN15252886). Registration date August 11, 2017.

### Study participants

Participants were recruited from October 2014 until May 2016, at the Academic Centre for Dentistry Amsterdam (ACTA). Patients referred to the Department of Periodontology with untreated periodontitis were selected based on the presence of radiographic bone loss of ≥1/3 of the root length on minimum 2 non-adjacent teeth, on peri-apical long cone radiographs. Control subjects were screened for their periodontal condition using the Dutch Periodontal Screening Index (DPSI) [20]. Healthy controls were defined as subjects having a maximum probing depth of 4–5 mm in the absence of gingival recession (≤ DPSI 3-). Additionally, the absence of alveolar bone loss was confirmed on bitewing radiographs not older than 12 months. Subjects were excluded if they presented with a history of pathologic conditions that are known to systemically affect PMN numbers and function (such as hematological disorders, diabetes mellitus, antibiotics use within the last 6 months, recent history of illness or fever, allergies, alcoholism and pregnancy). Furthermore, individuals with less than 20 teeth, removable partial dentures, night guards, orthodontic banding, (peri) oral piercings or apparent oral lesions were also excluded.

### Bacterial culture

*Fusobacterium nucleatum* (*F.n.*) strain DSM 20482 was grown anaerobically (80% nitrogen, 10% carbon dioxide and 10% hydrogen) in brain-heart infusion (BHI) broth supplemented with 5 μg/ml hemin (Sigma-Aldrich Chemie B.V., Zwijndrecht, Netherlands) and 1 μg/ml menadione (Sigma). Bacteria were isolated from broth cultures by centrifugation, washed twice in sterile phosphate-buffered saline (PBS), prior to dilution with sterile PBS to give a final suspension of 4 × 10^8^ cells/ml which was stored at − 20 °C.

### Isolation and purification of PMNs

For each participant, the oPMNs and cPMNs were isolated and subsequently analyzed on the same day without delay. The collection procedure was based on previously described protocol [11]. All participants were instructed neither to gargle nor to clear their throat during the sampling procedure. The oPMNs were obtained by 4 serial rinses of the oral cavity with 20 ml of sterile sodium chloride solution (0.9% NaCl) for 30 s, with a 4½ min intermission. The pooled sample was kept on ice in a 50 ml centrifuge tube (Sigma) until the end of the collection procedure. One ml from the first rinse of each participant was pipetted into a 2 ml microtube and stored at − 80 °C for further analysis (see below *Protease activity*). Venous blood samples were drawn from the antecubital fossa of all subjects into 9 ml sodium heparin blood collection tubes (BD Vacutainer™, Breda, the Netherlands) and maintained at room temperature until the PMN isolation procedure. The isolation procedure of oPMNs and cPMNs was carried out as described before [11]. Cell counts were obtained using a Muse® Cell Analyzer (Merck Millipore, Darmstadt, Germany) and verified with a Bürker-Türk counting chamber and Trypan Blue exclusion for the viability of the PMNs.

### Analysis of PMN apoptosis

Cell death was analyzed by means of flow cytometry, using the commercially available apoptosis detection kit *(BD Pharmingen™ FITC Annexin V Apoptosis Detection Kit, BD Biosciences, San Diego, CA, USA)*. In brief, PMNs were incubated for 15 min in the dark at room temperature and fixated according to the manufacturer’s instructions*.* Flow cytometric analysis was performed within 1 h *on* a BD Accuri™ C6 flow cytometer (BD Biosciences) and the Accuri CFlow Plus software was used for data acquisition and analysis. The percentages of propidium iodide positive (PI^+^) PMNs were calculated, indicating apoptotic or damaged cells.

### Analysis of membrane-bound markers of PMN activation

PMN activation was analyzed for the expression of clusters of differentiation (CD) markers CD11b, CD63, and CD66b on the Accuri C6 flow cytometer (BD Biosciences). The low affinity immunoglobulin-Fcγ receptor IIIb (CD16b) was used as a PMN identification marker. PMNs were gated according to CD16 expression and the sideward scatter profile, and analyzed for the mAb of interest as described previously [12]. Briefly, PMNs were incubated on ice for 30 min with phycoerythrin (PE)-conjugated monoclonal antibodies anti-CD11b and anti-CD63 (BD Pharmingen, Breda, Netherlands) or fluorescein isothiocyanate (FITC)-conjugated monoclonal antibody anti-CD66b (BD Pharmingen) according to the manufacturer’s instructions. FITC and PE conjugated mouse IgG1 were used as isotype control antibodies with the same concentration as the specific antibodies. After incubation, PMNs were washed with PBS and resuspended in PBS containing 1% paraformaldehyde. CD16 positive PMNs were gated and analyzed for the expressions of the mAb of interest (CD11b, CD63, or CD66b). Data are expressed as mean fluorescence intensity (MFI) after correction for the non-specific binding of the isotype controls.

### ROS analysis

Non-stimulated samples were incubated for 30 min with 2 mM dihydrorhodamine123 (DHR) in PBS, in a shaking (50 rpm) waterbath at 37 °C. Stimulation was achieved by adding phorbol myristate acetate (PMA, Sigma) at a final concentration of 0.1 μg/ml or non-opsonized *F.n.* to the cell suspension in a ratio of PMN:*F.n.* of 1:20. Results were expressed as the MFI or the fold increase in MFI (the ratio of MFI of the PMA or *F.n.*-stimulated samples divided by the MFI of samples without stimulation).

### Protease activity

To quantify the protease activity in the oral rinses, one ml aliquots from the first rinses of each participant were retrieved from − 80 °C storage and thawed on ice. The protease activity was determined using black 96-wells microplates (F Bottom, Greiner Bio- One GmbH, Frickenhausen, Germany). Each microwell was filled with 70 ml of PBS, and 8 μM protease substrate PEK-054 ([FITC]-NleKKKKVLPIQLNAATDK-[KDbc]), a substrate for total protease activity [21]. As a positive control, trypsin from bovine pancreas was added in duplicate in two-fold serial dilutions, and sterile PBS was used as a negative control. Samples from periodontitis patients and controls were defrosted and 30 μl was added to each microwell. The increase in fluorescence was monitored over 60 min using a fluorescence microplate reader (Fluostar Galaxy, BMG Laboratories, Offenburg, Germany) with an excitation wavelength of 485 nm and an emission wavelength of 530 nm. Relative fluorescence (RF) values were obtained for periodontitis patients and controls. The total protease activity was defined in RF per Unit (RF U).

### Statistical analysis

All analyses were performed with the SPSS Statistics 23.0 software (IBM, Chicago, Illinois, USA). Means, standard deviations (SD), range and frequency distributions were calculated. Normal distribution of data was tested with the Kolmogoroff-Smirnov test and when needed, log transformation of data sets was performed before proceeding with parametric statistics. The characteristics of patient and control groups were compared with the Student’s T-test or the chi-square test, where appropriate. Within groups, comparisons between non-stimulated and stimulated samples (PMA or *F. nucleatum*) were performed using the Paired T-test. A further, linear regression analysis was performed to explore a possible relationship between the oPMN numbers and the measured PMN parameters. In these analyses, the log-transformed experimental data for oPMN counts were entered as dependent variables, and total protease activity was entered as the independent variable. A *P*-value < 0.05 was considered statistically significant.

## Results

### Study population

A total of 19 periodontitis patients and 16 control subjects were included in this study. A description of the characteristics of the participants is provided in Table 1. No significant differences were observed between the two groups in relation to age, sex distribution and number of teeth. Periodontitis patients were more often of non-Caucasian descent, smoked and had a higher body mass index (BMI) than the control group. Within the periodontitis group, an average of 9.8 teeth showed a radiographic bone loss of ≥50% of the root length.

**Table 1:** Characteristics of the study population stratified according to periodontal condition

	Control (*n* = 16)	Periodontitis (*n* = 19)	*p*-value
Age	44.1 ± 11.6	47.9 ± 12.4	0.354
Sex (male)	8 (50%)	11(55%)	0.765
Ethnicity (Caucasian)	12 (75%)	7 (37%)	**0.024**
Smoking (current smoker)	4 (25%)	11 (58%)	0.050
Body mass index	23.5 ± 2.2	27.9 ± 5.0	**0.006**
# of teeth	27.7 ± 3.0	26.6 ± 3.3	0.303
# of teeth with > 50% bone loss	0	9.8 ± 6.0	–
Counts (× 10^6^)			
cPMNs^a^	19.3 ± 11.2	17.6 ± 9.0	0.873
oPMNs^b^	1.8 ± 3.2	8.1 ± 11.5	**0.007**

Results for demographic, dental characteristics and counts of oPMNs and cPMNs. Values represent means ± standard deviations, or numbers (%) of subjects. *P*-values calculated by χ^2^-test or Student’s T-test; (n/a = not applicable). *P*-values < 0.05 were considered statistically significant and are shown in bold

^a^Total cPMNs obtained from a 6 ml tube of blood, after the isolation and purification steps

^**b**^Total oPMNs collected after 4 × 30 s rinsing with 4½ minutes intermission, after the isolation and purification steps

### Isolation of PMNs

The yield of cPMN from venous blood was comparable in healthy controls and periodontitis patients (mean 19.3 versus 17.6 × 10^6^, *P* = 0.873, Table 1). On average, 4.5 times more oPMN were retrieved from periodontitis patients (mean 8.1 × 10^6^ cells per sample) than from the control group (mean 1.8 × 10^6^ cells per sample, *P* = 0.007, Table 1).

### Apoptosis assay

**Fig. 1:**
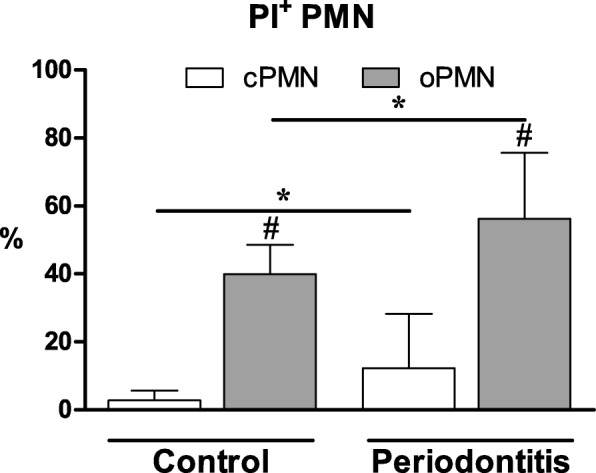
Apoptosis analysis of PMNs from controls (*n* = 16) and periodontitis subjects (*n* = 19) quantified by flow cytometry. The percentages of propidium iodide positive (PI^+^) PMNs were calculated, representing apoptotic cells. The different cell populations are expressed as a percentage of the total PMN cell population and are given as mean ± SD. *Comparisons periodontitis group versus control group, Student’s T-test, *P < 0.05. ^#^*Comparisons oPMN* versus *cPMN within subjects,* Paired T-test, ^#^*P* < 0.001

The apoptotic cPMNs (PI^+^) accounted for a mean of 2.8% in the control group. The periodontitis patients showed a higher mean percentage of PI^+^ cPMNs compared to the control group (mean 12.3%, *P* = 0.026, Fig. 1). Similar to the profile of the circulatory cells, the PI^+^ oPMNs percentages were higher in periodontitis patients (56.2%) than in controls (39.9%, *P* = 0.004, Fig. 1). Additionally, more PI^+^ cells were found amongst the oPMNs compared to the cPMNs in both patients and controls (*P* < 0.001, Fig. 1).

### Degranulation assay

**Fig. 2:**
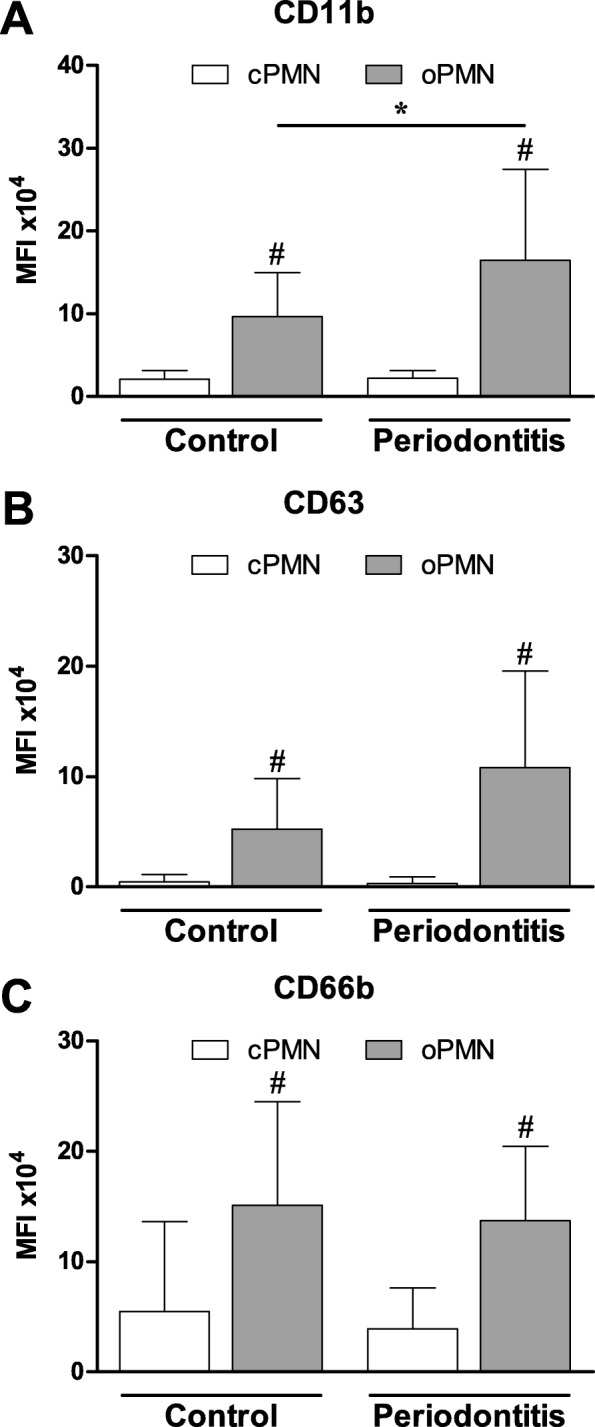
Mean fluorescence intensity (MFI) of cellular expression of (**a**) CD11b, (**b**) CD63 and (**c**) CD66b, on PMNs isolated from controls (*n* = 16) and periodontitis subjects (*n* = 19). PMNs were gated according to CD16 expression and the sideward scatter profile. Expressions of surface markers of interest were corrected for the non-specific binding of the isotype control antibodies. Data are mean ± SD. *Comparisons periodontitis group versus control group, Student’s T-test, *P = 0.024. ^#^*Comparisons oPMN* versus *cPMN within subjects,* paired T-test, all ^#^*P* < 0.001

The expression of CD11b, CD63, and CD66b on the cPMNs was low and comparable in healthy controls and in periodontitis patients (*p* > 0.05, Fig. 2a, b, and c, respectively). The oPMNs were more activated than cPMNs, as they expressed 3 to 36-fold higher levels of all three activation markers than the cPMNs, both in healthy controls and in periodontitis patients (all *P* < 0.001, Fig. 2). CD11b was more expressed by oPMNs derived from periodontitis patients than by oPMNs derived from controls (*P* = 0.024, Fig. 2a). The expression of CD63 and CD66b on the oPMNs was not statistically different between the periodontitis patients and controls (CD63 *P* = 0.054, Fig. 2b; CD66b *P* = 0.483, Fig. 2c).

### ROS assay

**Fig. 3:**
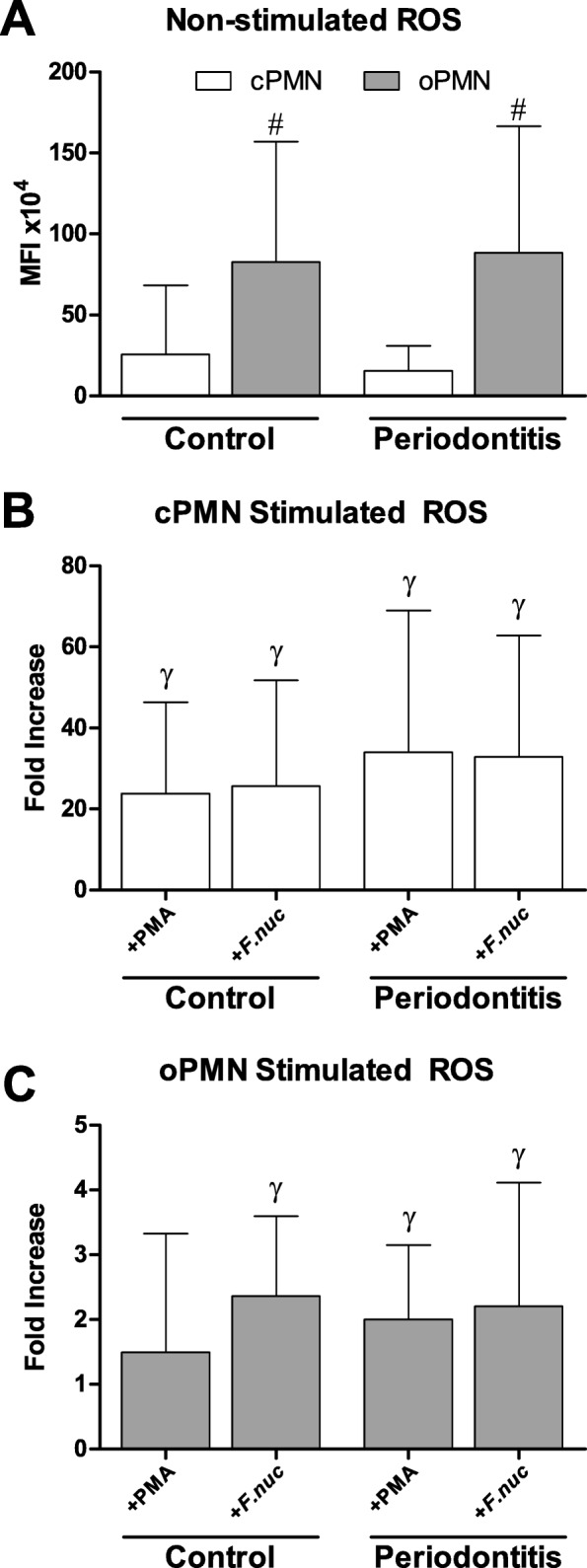
Mean fluorescence intensity (MFI) of reactive oxygen species (ROS) produced by PMNs isolated from controls (*n* = 16) and periodontitis subjects (*n* = 19). **a** Non-stimulated ROS production was calculated and expressed as mean ± SD. **b** Stimulated ROS production produced by cPMNs when incubated with phorbol myristate acetate (+PMA) or *F. nucleatum* (+*F.nuc*). **c** Stimulated ROS production produced by oPMNs when incubated with phorbol myristate acetate (+PMA) or *F. nucleatum* (+*F.nuc*). Ratios were labeled as fold increase and were calculated for the stimulated ROS samples (MFI of the PMA-stimulated or *F.n.*-stimulated samples, divided by the MFI of the non-stimulated ROS samples). ^#^*Comparisons oPMN* versus *cPMN within subjects,* Paired T-test, ^#^*P* < 0.001. ^γ^Comparisons to corresponding non-stimulated samples (panel **a**), Student’s T-test, all ^γ^*P* < 0.05

Non-stimulated cPMNs demonstrated comparable levels of ROS production between the control group and the periodontitis group (Fig. 3a). Upon stimulation with either PMA or *F. nucleatum*, ROS production from the cPMNs increased 24–26 fold for the control subjects (both *P* < 0.001, Fig. 3b), whereas the periodontitis patients showed an increase of 33–34 fold compared to the non-stimulated cPMNs (both *P* < 0.001, Fig. 3b). The same pattern was observed for the oPMNs’ non-stimulated and stimulated ROS production levels. Comparable ROS levels were produced by oPMNs originating from controls and periodontitis patients (Fig. 3a and c). The oPMNs non-stimulated ROS levels were 3–5 higher compared to the non-stimulated ROS levels produced by cPMNs (both *P* < 0.001, Fig. 3a). Upon stimulation with PMA, only the oPMNs’ from the periodontitis group showed a significant increase of ROS production (2 fold increase, *P* = 0.012, Fig. 3c), while ROS levels were not significantly induced by PMA in the control group. Upon bacterial stimulation with *F. nucleatum*, oPMNs from both the control group and the periodontitis group produced higher levels of ROS (control 2.4 fold increase, *P* < 0.001; periodontitis 2.2 fold increase, *P* = 0.038, Fig. 3c).

### Protease activity

**Fig. 4:**
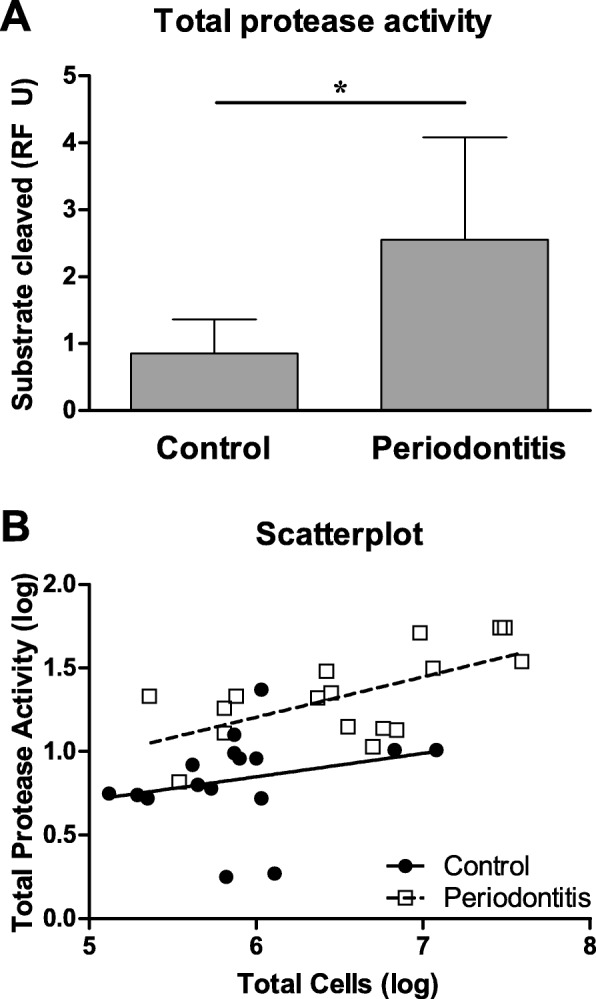
Total protease activity measured from oral rinses originating from controls (*n* = 16) and periodontitis subjects (*n* = 16). **a** Total protease activity was measured as relative fluorescence per unit (RF U) and expressed as mean ± SD. *Comparisons periodontitis group versus control group, Student’s T-test, *P < 0.001. **b** Total protease activity was only predicted by oPMN counts in periodontitis patients (dashed line, r^2^ = 0.3989, *P* = 0.0065) and not in the control group (straight line, *r*^*2*^ = 0.0629, *P* = 0.3486)

The total protease activity was analyzed from the first rinse samples and showed a 3.1 times higher activity in the periodontitis group compared to the control group (*P* < 0.001, Fig. 4a). Additionally, the number of oPMNs was a significant predictor for the total protease activity (*r*^2^ = 0.386, *P* < 0.001). Further subanalysis revealed that this relation was only found amongst the periodontitis patients (*r*^2^ = 0.399, *P* = 0.007, Fig. 4b), and not in the control subjects (*r*^2^ = 0.063, *P* = 0.349, Fig. 4b).

## Discussion

The purpose of the current investigation was to study the numbers and function of the oPMNs in subjects with periodontitis. Normally, oPMNs contribute to oral health maintenance and form a part of the innate oral defense mechanisms. However in periodontitis these cells may be left with limited or depleted functional capabilities. The results of the present study showed a significant higher number of oPMNs in patients with untreated periodontitis compared to subjects without periodontitis. Confirming our previous results [11], the oPMNs showed increased expression of degranulation markers and unstimulated ROS release compared to the cPMNs; this was observed in both the periodontitis and control subjects. However, the periodontitis patients showed more apoptotic oPMNs and increased degranulation compared to the control group. Combined, these findings suggest that as the PMNs migrate through oral tissues and “travel” along the biofilms surrounding the teeth, these cells age, use their granule resources and lose ROS production capabilities; these traits are more pronounced in periodontitis.

On average, we found 4.5 times more oPMNs in the patient group. Our results confirm previous reports of increased PMN numbers in the presence of gingival inflammation and deepened pockets [4, 5, 7, 13, 18]. Higher numbers of oPMN in periodontitis is most likely explained by an increased periodontal inflamed surface area (PISA), which has been reported to reach 5–20 cm^2^ [22, 23]. In response to the inflamed oral surroundings, PMNs leave the blood circulation, enter the periodontal space and mix in the oral fluid. Moreover, as the oral biofilm acquires pathogenic traits, more chemokines and cytokines are released into the periodontal tissues, resulting in increased recruitment of PMNs [24, 25].

We found more apoptotic oPMNs in periodontitis patients compared to controls. One explanation for this finding might be that there are more cPMNs being programmed for apoptosis in periodontitis and that these cells appear in patients’ rinse samples. We suggest that more apoptotic cPMN could be a systemic characteristic of the periodontitis patients, and therefore also found in the oral cavity. More apoptotic oPMN in periodontitis is in contrast to the recent results of Lakschevitz and co-workers who suggested lower numbers of apoptotic oPMN in periodontitis compared to controls [8]. In our protocol, the participants repeated the oral rinsing 4 times, while the subjects from Lakschevitz et al. rinsed 6 times consecutively [8]. In pilot experiments preceding the current study, we observed that the viability of oPMNs increased by 17.7% between the first rinse and the fourth successive rinse, suggesting that fresh, new PMNs are retrieved as the oral rinse is repeated (data not shown). Furthermore, Lakschevitz et al. based their conclusions about oPMN viability on a small sample subset (*N* = 3, for this sub-analysis) [8], whereas we analyzed nineteen periodontitis patients and observed inter-individual differences.

Upon PMN activation, the cytoplasmic granules fuse with the PMN cellular membrane and an upregulation of the granular markers (CD11b, CD63 and CD66b) becomes measurable. The increased expression of these CD markers in our study confirms that the oPMNs have undergone migration and degranulation relative to the cPMNs. Additionally, the upregulated expression of CD11b and the tendency for increased CD63 in periodontitis patients’ oPMNs, suggest a higher release of granular content, possibly related to activation during the migration through the inflamed periodontal tissues and along the subgingival biofilm. Important to note is that periodontal inflammation can be present in various inflammatory states, ranging from a pre-inflammatory state within stressed tissues, to a full-fledged inflammatory state within damaged tissues. The results from this study corroborate the recent findings by Fine et al., who also found degranulated oPMNs in periodontitis [18].

Intrinsic increase of ROS production by cPMN has been proposed for patients with chronic periodontitis as a susceptibility trait [26–29]. In our study, we could not confirm this trait, since our results showed similar unstimulated and stimulated ROS levels originating from periodontitis patients and healthy controls. A plausible explanation for the discrepancy with the literature could be the application of different methods used to analyze ROS production. While most studies have used luminol enhanced chemi-luminescence for the measurement of total ROS generation (intra- and extracellular), our study has used flowcytometric analysis. In contrast to the chemi-luminescence method, which measures various ROS species in a multi-well assay, the flowcytometric analysis used in this study detects only hydrogen peroxide at a single cell level. However, the flowcytometric method is preferred because it is applicable in situations with low cell density, such as in our oral rinse samples.

Another possible reason may lie in the socio-demographic differences between study populations. Matthews et al. demonstrated that when periodontitis patients were compared with age-, and sex-matched controls, only the unstimulated cPMNs produced higher levels of ROS, while the stimulated levels were comparable between the measured groups [29]. In the present study, age and sex distribution were not different between the periodontitis and control subjects, however the ethnicity, BMI and smoking frequencies were different. Ethnic background can influence PMN numbers and ROS activity [30, 31]. Furthermore, it has been suggested that PMNs in obese subjects may be in a primed state compared to non-obese subjects, and can participate in the pathogenesis of obesity-related diseases, such as periodontitis [32]. Another study demonstrated that cigarette smoke extract could lower the PMN ROS production capabilities in response to *F. nucleatum* specifically [33].

Our group previously evaluated ROS levels of oPMNs and/or cPMNs in response to *F. nucleatum* [11, 12, 34]. Several PMN receptors are involved in active ROS production, like Toll-like receptors and protein kinase C agonists, from which Toll-like receptors are the most efficient in ROS generation [35, 36]. While PMA stimulation acts via protein kinase C, *F. nucleatum* is able to activate the PMNs’ Toll-like receptors (TLR-2, TLR-4, TLR-9) [30]. *F. nucleatum* stimulation may therefore result in a different oPMN ROS response than PMA stimulation. The ROS levels that were recently reported in chronic periodontitis patients [18] were acquired using an extensive flowcytometric gating strategy in order to identify PMN sub-populations. Our study did not evaluate the PMNs to this extent. Fine and co-workers observed that PMA stimulation did not significantly induce ROS levels in chronic periodontitis patients and suggested that oPMNs from these subjects might have exhausted their ROS potential. Interestingly, the same results were observed in the control subjects in the current study, whereas we showed that ROS levels from the oPMNs isolated from periodontitis patients were significantly enhanced after stimulation. ROS production is known to show inter-individual variations and is stimulus dependent. In addition, the inflammatory state of the participants in our study may not be exactly identical to that of the participants from previous reports regarding ROS production in chronic periodontitis. We presume that the general inflammatory status of an individual (high – low) is reflected by the cPMNs, which does not necessarily reflect the oral inflammatory status. In this line, the oPMNs are a better reflection of the oral local environment than the cPMNs.

In addition to the biological processes within the periodontal environment, the high numbers of oPMNs in the oral cavity in periodontitis patients may also have consequences for the integrity of the oral mucosa. The increased protease levels that were observed in the periodontitis group were also positively correlated to the number of oPMNs. The combination of increased numbers, with unrestrained excessive ROS release and proteolytic enzymes, can negatively influence the balance of the healthy/normal oral ecology within an individual. As such, oxidative damage and degradative enzymes may contribute to the increasing vascular permeability [37, 38] and a continuous and excessive efflux of the PMNs into the oral cavity in untreated periodontal disease may contribute to collateral tissue damage not only within the periodontium, but also affect the integrity of the oral mucosa in these patients. Consequently these changes make the oral mucosa more vulnerable to environmental challenges including components of cigarette smoke, and metabolites of alcohol such as acetaldehyde and acetate.

## Conclusions

In periodontitis, one can expect higher numbers of oral PMNs than in health. These cells are more degranulated and more often apoptotic than oPMNs from non-periodontitis individuals. We suggest that the primed cPMN is a systemic trait in periodontitis patients, showing an increased proportion of apoptotic cells compared to controls. This observed PMN characteristic could hamper their proper function in the periodontal tissues and their surveillance function in the oral cavity as a whole. Nevertheless, the oPMNs in periodontitis display sufficient functionality as shown by their responsiveness (ROS production) after ex vivo stimulation. We therefore suggest that the oPMNs in periodontitis participate in the maintenance of the oral ecosystem. However, the question remains whether in periodontitis, the increased oPMN counts, increased release of granule content, and excessive ROS and proteases can be detrimental to the oral mucosa integrity.

## Abbreviations

BHI: Brain-heart infusion
BMI: Body mass index
CD: Clusters of differentiation
cPMN: Polymorphonuclear neutrophils isolated from the blood circulation
DHR: Dihydrorhodamine123
*F.n.*: *Fusobacterium nucleatum*
FITC: Fluorescein isothiocyanate
MFI: Mean fluorescence intensity
oPMN: Polymorphonuclear neutrophils isolated from the oral cavity
PBS: Phosphate-buffered saline
PE: Phycoerythrin
PI: Propidium iodide
PISA: Periodontal inflamed surface area
PMA: Phorbol myristate acetate
PMN: Polymorphonuclear neutrophils
ROS: Reactive oxygen species
